# The gut microbiota: a major player in the toxicity of environmental pollutants?

**DOI:** 10.1038/npjbiofilms.2016.3

**Published:** 2016-05-04

**Authors:** Sandrine P Claus, Hervé Guillou, Sandrine Ellero-Simatos

**Affiliations:** 1 Department of Food and Nutritional Sciences, The University of Reading, Reading, UK; 2 Toxalim, UniversitÃ© de Toulouse, INRA, Toulouse, France

## Abstract

Exposure to environmental chemicals has been linked to various health disorders, including obesity, type 2 diabetes, cancer and dysregulation of the immune and reproductive systems, whereas the gastrointestinal microbiota critically contributes to a variety of host metabolic and immune functions. We aimed to evaluate the bidirectional relationship between gut bacteria and environmental pollutants and to assess the toxicological relevance of the bacteria–xenobiotic interplay for the host. We examined studies using isolated bacteria, faecal or caecal suspensions—germ-free or antibiotic-treated animals—as well as animals reassociated with a microbiota exposed to environmental chemicals. The literature indicates that gut microbes have an extensive capacity to metabolise environmental chemicals that can be classified in five core enzymatic families (azoreductases, nitroreductases, β-glucuronidases, sulfatases and β-lyases) unequivocally involved in the metabolism of >30 environmental contaminants. There is clear evidence that bacteria-dependent metabolism of pollutants modulates the toxicity for the host. Conversely, environmental contaminants from various chemical families have been shown to alter the composition and/or the metabolic activity of the gastrointestinal bacteria, which may be an important factor contributing to shape an individual’s microbiotype. The physiological consequences of these alterations have not been studied in details but pollutant-induced alterations of the gut bacteria are likely to contribute to their toxicity. In conclusion, there is a body of evidence suggesting that gut microbiota are a major, yet underestimated element that must be considered to fully evaluate the toxicity of environmental contaminants.

## Introduction

The human gut microbiota is a dynamic ecosystem formed by a pool of 400–1000 adherent and non-adherent bacterial species belonging mostly to two dominant phyla, the Firmicutes and the Bacteroidetes.^1^ Although the composition of an adult microbiota remains relatively stable, it is well known that the microbial diversity is acquired very early in life within the first hours post birth, and is shaped over time as the diet becomes more complex and the immune-system matures. Hence, the combination of multiple factors including genotype, mode of delivery, early antibiotic therapy, diet composition, lifestyle, social interactions and environmental exposure to various xenobiotics shape the gut microbiota to make every individual microbially unique.^2,3^ This is of importance because the gut microbiota fulfills many critical roles in essential host functions such as protection against pathogens, immune-system modulation, fermentation of non-digestible dietary fibres, anaerobic metabolism of peptides and proteins, interaction with the host’s circadian clock and biotransformation of xenobiotics.^4–6^ Such a complex symbiotic interaction is the result of a remarkable metabolic activity driven by a genetic pool whose size is a hundred times larger than the human one. Alterations of the microbiota composition (called dysbiosis) and/or optimal functions are associated with various prevalent metabolic and immune diseases, including obesity,^7^ inflammatory bowel disease,^8^ diabetes,^9^ hepatic diseases,^10,11^ Crohn’s disease,^12^ colorectal cancer^13^ and allergy.^14^

**Figure 1:**
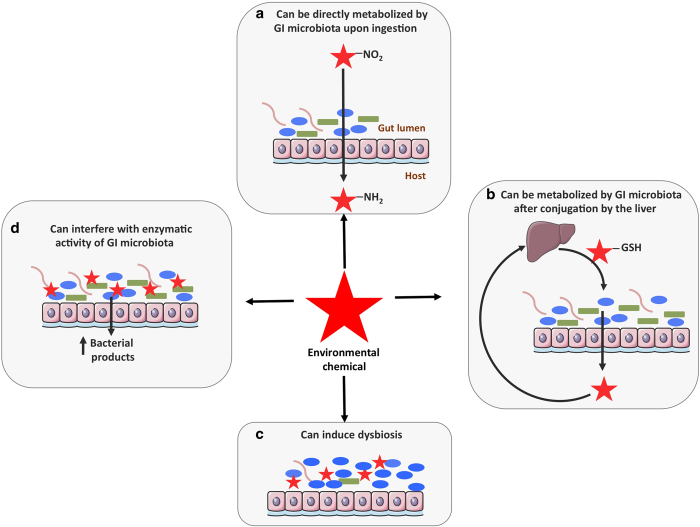
Environmental chemicals and the GI microbiota interact via multiple mechanisms. (**a**) Environmental chemicals that are poorly absorbed after ingestion are swept to the distal small intestine and caecum by peristalsis, and those that partition across the intestinal wall from the blood may be directly metabolised by the GI microbiota. (**b**) Most xenobiotics are non-polar and therefore readily absorbed from the GI tract, then transported by the portal blood to the liver for detoxification. The liver tends to oxidise xenobiotics, forming conjugates with glucuronic acid, sulfate, or glutathione that can be excreted in the bile and enter the intestine where microbiota metabolism can take place. The GI microbiota generally deconjugates and reduces the hepatic xenobiotic metabolites, resulting in the formation of non-polar molecules of lower molecular weight, which are readily reabsorbed. Microbiota-mediated deconjugation of metabolites previously conjugated by the liver may regenerate the original xenobiotic or form new toxic metabolites. (**c**) Environmental chemicals can also interfere with the composition of the GI microbiota, which may lead to detrimental consequences for the host. (**d**) Pollutants can also change the metabolic activity of the GI microbiota, which may affect the activity of endogenous metabolites or the toxicity of other xenobiotics that depend on gut bacteria for their metabolism.

Synthetic chemicals currently used for diverse industrial and agricultural applications are leading to widespread contamination of the environment and their effects on human health are a global concern. Mounting evidence indicates that exposure to these environmental chemicals is one of the multiple environmental factors contributing to the development of several health disorders. *In vitro*, *in vivo* and epidemiological studies have, for example, linked human exposure to endocrine-disrupting chemicals to obesity, metabolic syndrome and type 2 diabetes.^15^ However, it is unclear how the gastrointestinal (GI) microbiota and environmental chemicals interact and whether these interactions are relevant for human health. In a recent review, it was suggested that GI microbes might affect obesity and diabetes by altering the absorption, disposition, metabolism and excretion of environmental chemicals.^16^ Here we aimed to describe how environmental chemicals and GI microorganisms might interact and to evaluate the toxicological relevance of these interactions. We identified four different types of interactions, illustrated in Figure 1: first, the GI microbiota can metabolise a variety of environmental chemicals, directly upon ingestion (Figure 1a) or after their conjugation by the liver (Figure 1b). Reciprocally, environmental chemicals can interfere with the composition (Figure 1c) and/or metabolic activity (Figure 1d) of the GI microbiota, with potentially deleterious consequences for the host. Table 1 presents information on the levels of exposure of the various compounds that are presented in this review and briefly summarises their interaction with the gut microbiota.

## The GI microbiota can metabolise environmental chemicals

GI microorganisms have been known for decades to be involved in the biotransformation of xenobiotics. Back in 1973, Scheline suggested that the potential of the GI microbiota to metabolise foreign compounds was at least as great as that of the liver.^17^ More than 40 drug substrates have since been identified for the GI microbiota,^5,18–21^ highlighting the ability of gut microbes to perform diverse chemical transformations on drugs, including reduction, hydrolysis, the removal of a succinate group, dehydroxylation, acetylation, deacetylation, the cleavage of a N-oxide bounds, proteolysis, denitration, deconjugation, thiazole ring opening, deglycosylation and demethylation. Haiser and Turnbaugh recently pointed out the relevance of the bioremediation literature; particularly as concerns the metabolism of environmental chemicals:^20^ a catalogue of microbial biocatalytic reactions on environmental pollutants currently lists almost 1,500 reactions carried out by 529 microorganisms affecting some 1,369 compounds.^22^ The GI microbiota is thought to be less diverse than that of soil environments,^23^ but these findings nevertheless suggest that the GI bacteria may have a significant, but underestimated, capacity to metabolise environmental chemicals.

The GI tract is the main route by which xenobiotics enter the human body. The rate and extent of bacterial metabolism is influenced by the amounts of xenobiotics reaching the distal gut, where bacteria concentration is maximal. Environmental chemicals may be poorly absorbed after ingestion, subsequently being swept to the distal small intestine and caecum by peristalsis. Alternatively, they or their metabolites may partition from the blood across the intestinal wall. Consequently, a number of chemicals are directly metabolised by the GI microbiota (Figure 1a). Environmental chemicals (or their metabolites) may also be excreted in the bile. Most xenobiotics are non-polar and are therefore absorbed in the GI tract and transported by the portal blood to the liver for detoxification. The liver generally oxidises xenobiotics and produces glucuronic acid, sulfate or glutathione conjugates. In most cases, conjugation reactions facilitate excretion and conjugates are eliminated in urine. However, conjugates can also be excreted in the bile. The factors that determine whether a chemical is excreted into bile are not fully understood, a general rule being that low-molecular-weight compounds (<325 kDa) are poorly excreted into bile, whereas compounds with higher molecular weight (>325) can be significantly excreted.^24^ Conjugates secreted into bile enter the small intestine where their absorption from the GI tract is highly variable. Those that are not absorbed move down to the large intestine where they may be metabolised by the microbiota (Figure 1b). The GI microbiota tends to deconjugate and reduce hepatic xenobiotic metabolites, resulting in the formation of non-polar molecules of lower molecular weight, which are readily reabsorbed. The reabsorption of these non-polar molecules and their return to the liver are referred to as ‘enterohepatic circulation’. Enterohepatic circulation controls the storage and reuse of endogenous substrates in the body, such as the bile acids and steroids. However, it also delays the elimination of environmental chemicals from the body.

**Figure 2:**
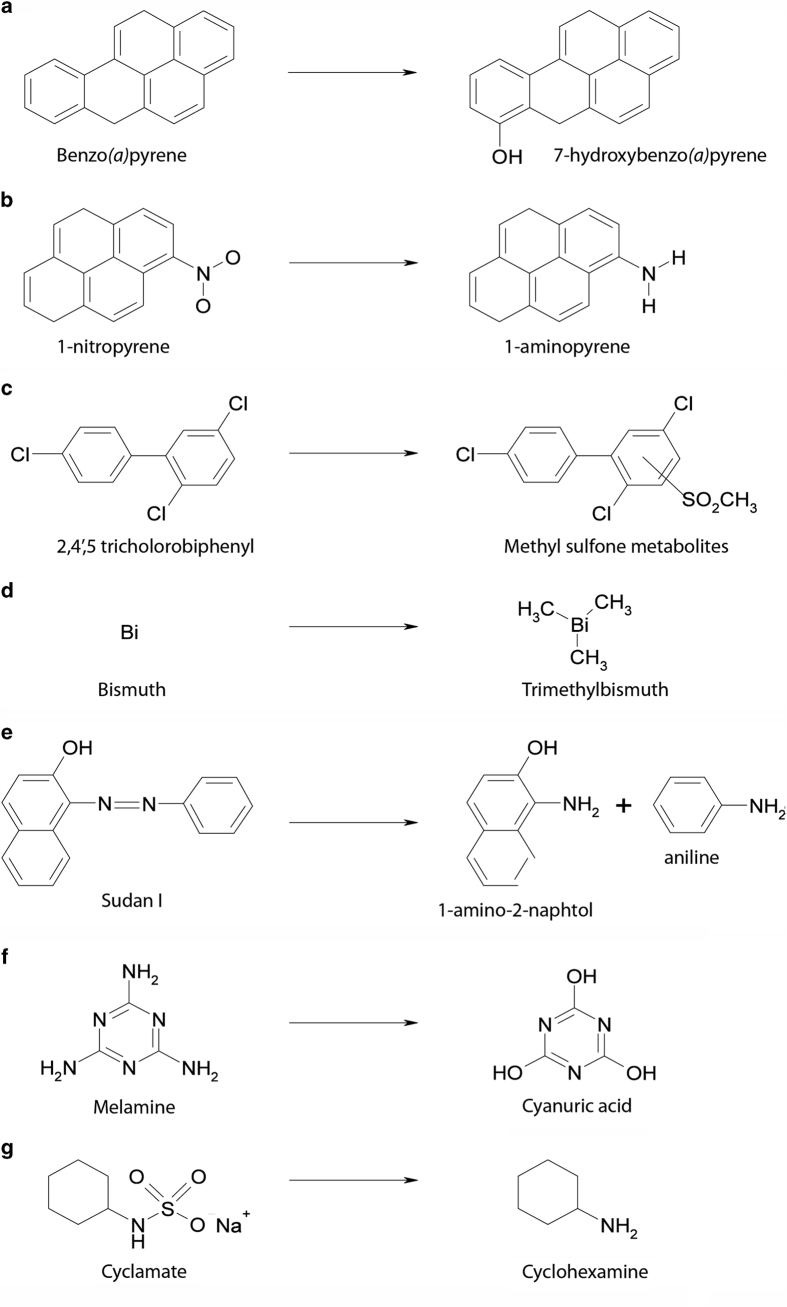
Examples of GI-bacteria-mediated transformations of environmental xenobiotics.

In this section, we will briefly present the xenobiotic-metabolising capabilities of the GI microbiota. We will then present some of the environmental pollutants identified as substrates of the GI microbiota. We consider these pollutants by chemical class and discuss the toxicological relevance of GI microbiota-mediated metabolism for each chemical. As it is not possible to discuss all xenobiotics in details in this review, Supplementary Table 1 provides a more exhaustive list of substrates derived from the interaction of the gut microbiota with environmental pollutants. In addition, Figure 2 presents examples of environmental xenobiotics and their gut-microbiota-derived metabolites.

### Xenobiotic-metabolising enzymes of the GI microbiota

**Figure 3:**
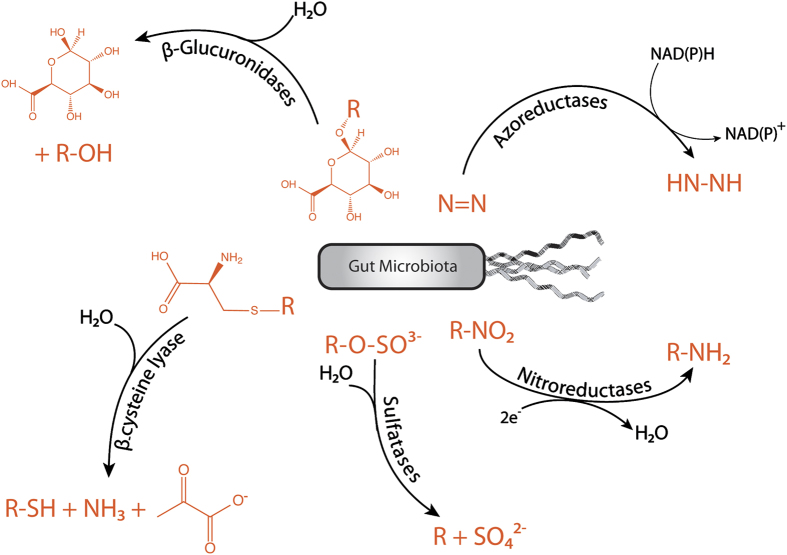
Xenobiotic-metabolising enzymes of the GI microbiota: (i) The reductive cleavage of azo (N=N) bonds is performed by bacterial azoreductases. Three groups of azoreductases have been described: flavin-dependent NADH preferred azoreductases, flavin-dependent NADPH preferred azoreductases and flavin-free NADPH preferred azoreductases. (ii) Bacterial nitroreductases reduce nitro (–NO_2_) functional groups to the corresponding amines. Two types of bacterial nitroreductases have been described: type 1 nitroreductases are oxygen-insensitive and catalyse the sequential reduction of nitro groups through the addition of electron pairs from NAD(P)H to produce the nitroso, hydroxylamino and amino derivatives. Type 2 nitroreductases are oxygen-sensitive and catalyse the single-electron reduction of the nitro group to produce a nitro anion radical. (iii) Endogenous sulfate esters are hydrolysed in the GI tract by sulfatases of bacterial origin. (iv) Glutathione conjugates of xenobiotics are also extensively excreted in the bile. They are degradated by various mammalian enzymes (γ-glutamyl transpeptidase and carboxypeptidase), resulting in the formation of cysteine conjugates. These cysteine conjugates may reach part of the GI tract that contain ß-lyase activity and be converted to their corresponding thiol. (v) ß-glucuronidases are present throughout the GI tract and play a role in the hydrolysis of xenobiotic glucuronides, the largest class of xenobiotic conjugates excreted in bile. The intestinal microorganisms are thought to be the major source of ß-glucuronidase because hydrolysis of many xenobiotic glucuronides is dramatically reduced (>90%) in GF or antibiotic-treated rats.

Although various chemical reactions have been attributed to GI microorganisms, only a few enzyme families have been identified for GI microbial xenobiotic metabolism; mainly azoreductases,^25,26^ nitroreductases,^27^ β-glucuronidases,^28^ sulfatases^29^ and β-lyases^30^ (Figure 3).

### A variety of environmental chemicals are metabolised by the GI microbiota

#### Polycyclic aromatic hydrocarbons (PAHs)

PAHs are among the most widespread organic pollutants generated by the incomplete combustion of carbon-containing fuels and are found in tobacco smoke, urban-air particulates, diesel exhaust and some food products, such as grilled and smoked meat. The toxicity of PAHs is structure dependent; some have oestrogenic properties and some have been classified as probable human carcinogens. Adult exposure to PAHs has been associated with higher risks of lung and bladder cancer.^31^ Van de Wiele *et al.* evaluated the estrogenicity of four PAHs (naphthalene, phenanthrene, pyrene and benzo(a)pyrene) before and after digestion by a typical human microbiota *in vitro.*^32^ Although the parent PAH molecules were not oestrogenic, the colonic digests displayed a significant oestrogenic activity. Hydroxy-PAHs, in particular 1-OH pyrene and 7-OH benzo(a)pyrene, were identified as the oestrogenic metabolites. This suggests that the microorganisms present in the human colon can bioactivate PAHs, by converting them to oestrogenic molecules. In addition, it has been shown that rat and human gut microbiota could regenerate benzo(a)pyrene from its hepatic conjugate, reversing the endogenous detoxification process, which is of potential toxicological relevance.^33^

#### Nitrated PAHs or nitro-PAHs

Nitro-PAHs are formed by the nitration of PAHs. Humans are mainly exposed through inhalation of nitro-PAHs present in urban-air particulate matter and diesel fuel emissions. Nitro-PAHs display a broad spectrum of mutagenic, genotoxic and carcinogenic properties. They are generally extensively metabolised by the human organism with significant involvement of the GI microbiota.^34^

2-nitrofluorene (NF) is one of the dominant nitro-PAHs in the environment and will be discussed further as a model compound for nitro-PAHs. *In vitro*, incubation of human faeces with NF removed the direct-acting mutagenicity of NF.^35^ *In vivo*, NF ingested by conventional rats is reduced to 2-aminofluorene by the intestinal bacteria, acetylated and further hydroxylated in the liver. This metabolic route is quantitatively the most important and results in the formation of hydroxylated 2-acetylaminofluorene.^36^ An alternative metabolic route results in the formation of hydroxylated nitrofluorenes, which are responsible for direct-acting mutagenicity.^36^ Comparison of NF metabolism in GF versus conventional rats showed that, after ingestion, the mutagenic potential of urine derived from GF animals was 6 times higher than the one derived from conventional animals.^36^ It was shown that this effect was mainly due to the presence of hydroxylated-NF and the absence of hydroxylated acetylaminofluorene.^36^ Therefore, GI bacteria appear to protect their host from the formation of mutagenic metabolites of NF. However, when comparing the potential of NF to form DNA adducts in mice with or without microbiota (conventional mice, mice re-conventionalised with mice faecal suspension or mice colonised with human faecal suspension *versus* GF mice), none were observed in GF mice, whereas DNA adducts were detected in the liver, colonic epithelium and kidney of all groups of mice hosting a microbiota.^35^ DNA adduct formation is regarded as a critical event in the development of cancer and may be used as an *in vivo* biomarker of cancer risk. This GI microbiota-dependant increase in DNA adduct formation might be related to the nitroreduction of NF by the bacteria, since 2-acetylaminofluorene is a potent carcinogen known to promote DNA adducts.^37^

#### Nitrotoluenes

Nitrotoluenes are important intermediates in the manufacture of dyes and plastics. 2-nitrotoluene is genotoxic, and 2,6-dinitrotoluene is hepatocarcinogenic in rats.^38,39^ After a single oral dose of labelled 2,4-dinitrotoluene, conventional rats excreted four major metabolites: 2,4-dinitrobenzoic acid and 2,4-dinitrobenzyl glucuronide, resulting from oxidation and oxidation followed by glucuronidation of the parent compound, respectively, and 4-*N*-acetyl-2-nitrobenzoic acid and 2-amino-4-nitrobenzoic acid, as a result of nitroreduction and oxidation, respectively. These last two metabolites were barely detectable in GF rats, suggesting that the GI microbiota is largely responsible for the nitroreduction of 2,4-dinitrotoluene.^40^ Human faecal samples can also reduce 2,4-dinitrotoluene.^41^ However, the genotoxicity of 2-nitrotoluene and 2,6-dinitrotoluene is due to a product of (di)nitrobenzyl alcohol reduction rather than from reduced metabolites and the gut bacteria plays an important role in this toxicity: both 2-nitrotoluene and 2,6-dinitrotoluene are absorbed in the small intestine and metabolised by the liver to 2-nitrobenzyl alcohol and 2,6-dinitrobenzyl alcohol. These compounds are conjugated with glucuronic acid before excretion in the bile. The GI microbiota then hydrolyses the glucuronide bound and reduces one or both of the nitro groups. The amino derivatives are then reabsorbed and oxidised in the liver where they covalently bind to DNA.^39^

#### Pesticides

Pesticide is a generic term that includes herbicides, fungicides, insecticides and antimicrobials. Thousands of different chemicals are used as pesticides, and human exposure to these pesticides is widespread. The most frequently used chemical families are organochlorines, organophosphates, carbamates, triazines and pyrethroids. Little is known about the role of the gut microbiota on their metabolism. Yet, several chemicals have been associated with bacterial metabolism.

Dichlorodiphenyltrichloroethane (DDT) is an organochlorine insecticide currently banned in most developed countries. However, DDT is highly persistent and contamination is still widespread. DDT displays oestrogenic and antiandrogenic activity in various tissues.^42^ DDT exposure has been associated with increased risk of breast, liver and testicular cancers^43^ and metabolic diseases.^15^ Rat and human GI faeces can metabolise DDT to dichlorodiphenyl-dichlorophenylethane (DDD).^44,45^ *In vivo*, DDD was detected in rats receiving DDT by stomach tube and not in those receiving DDT intraperitoneally,^44^ suggesting probable involvement of the GI microbiota in DDT metabolism. It remains unclear whether this biotransformation corresponds to bioactivation or detoxification, as both DDT and DDD are probable endocrine disruptors in humans.

Propachlor is an acetamide herbicide that has not been manufactured since 1998. Long-term propachlor treatment-induced tumours at single sites in rats.^46^ Propachlor is metabolised by the rat GI microbiota following the biliary excretion of glutathione and cysteine conjugates of the parent compound.^47,48^ Glutathione conjugation protects against propachlor-induced cytotoxicity in hepatocytes,^49^ so the deconjugation of conjugates by GI microbiota would be expected to increase propachlor toxicity *in vivo*.

#### Polychlorobiphenyls

PCBs form a group of 209 highly persistent chemicals. More than one million tons of technical PCB mixtures have been produced worldwide since their first commercial use in the late 1920s. Environmental exposure to PCBs has been associated with an increased risk of breast cancer,^50^ adverse reproductive outcomes,^51^ delayed neurodevelopment,^52^ impairment of the immune system^53^ and metabolic disruptions.^42^ The manufacture, processing and distribution of PCBs have thus been prohibited in most industrialised countries since the late 1980s, but they are still being released into the environment, through inappropriate disposal practices or leaks in electrical equipment and hydraulic systems. Human exposure to PCBs occurs mostly via ingestion of contaminated food, but also via inhalation and dermal absorption. The first step in the metabolism of PCBs is activation by oxidation catalysed by hepatic cytochromes P450, leading to the formation of an arene oxide intermediate. Two further metabolic pathways have been reported in mammals: hydroxylation; yielding biphenylols; and metabolism through the mercapturic acid pathway, which generates methyl sulfone (MeSO_2_) metabolites.^54^ The major metabolic route is hydroxylation generally followed by excretion. Nevertheless, significant amounts of MeSO_2_ metabolites of PCBs accumulate in tissues: the arene oxide intermediate is conjugated to glutathione, the glutathione conjugate is cleaved to yield a PCB–cysteine conjugate, which can be further cleaved by a bacterial C–S-lyase enzyme, leading to the formation of a PCB thiol. This thiol can be methylated to a PCB methyl sulfide (MeS-PCB) in the GI tract, absorbed and oxidised to the corresponding MeSO_2_-PCB in the liver. The gut microbiota thus has an important role in the formation of MeSO_2_-PCB.^55^ When GF and conventional rodents were injected with labelled 2,4’,5-trichlorobiphenyl, the level of radioactivity was 15 times higher in the adipose tissue,^48^ lung, kidney and liver^56^ of conventional animals, due to the accumulation of MeSO_2_-triCB. Bacteria-mediated metabolism of PCBs to MeSO_2_-PCBs is toxicologically relevant as they bind to specific proteins and accumulate in lipophylic tissues.^54^ In humans, they accumulate in the liver, lungs and adipose tissue and may be responsible for the chronic lung dysfunction symptoms observed in a mass food-poisoning incident.^57^

#### Metals

Many metals, such as mercury, lead, cadmium and arsenic are potent toxicants to living organisms. The chemical form of a metal strongly influences its rate of absorption and excretion and its distribution in the tissues in which it exerts its biological effects. Studies of the interconversion of the different forms of a metal are therefore crucial to evaluate its possible effects on health.^58^

Mercury may occur as an element and as inorganic and organic compounds, all of which have different toxicological properties. However, organic compounds of mercury are frequently the most toxic, with methyl mercury the principal source of organic mercury in humans. Contamination occurs through ingestion of contaminated fish and other aquatic food. *In vitro*, the incubation of methyl mercuric chloride with rat, mouse and human faeces, results in the production of elemental mercury.^58,59^ *In vivo*, the importance of bacterial demethylation for the elimination of mercury has been confirmed in studies where the suppression or absence of the GI microbiota was associated with lower faecal excretion of total mercury^60–62^ and with increased accumulation of mercury in most tissues, including the brain, the principal site of methyl mercury toxicity.

Arsenic is a ubiquitous environmental contaminant present in its trivalent or pentavalent state in both organic and inorganic compounds. Human exposure occurs primarily by consumption of contaminated fish and crustaceans. Chronic exposure is associated with the development of bladder, liver, kidney and lung cancers.^63^ The reduction of arsenic acid (iAsV) to arsenous acid (iAsIII) by rat caecal bacteria has been reported.^64^ In human, iAs is sequentially methylated and predominantly excreted as dimethylarsinic acid. This methylation process was originally considered to be a detoxification process but the formation of highly reactive methylated intermediates (monomethylarsonous acid MMAIII and dimethylarsinous acid DMAIII) has led to reconsider methylation as an activation process. Rodent and human gut microbes methylate iAs to monomethlyarsonic acid (MMAV), monomethlyarsonous acid (MMAIII) and monomethylmonothioarsonic acid (MMMTAV).^64–66^ The methylation of arsenic by GI bacteria has long been thought to have a small contribution to the overall methylation process *in vivo*, because iAsV and iAsIII are rapidly absorbed in the small intestine. However, soil- and/or dietary-bound arsenic may be digested differently: Van de Wiele *et al.* have shown that human colonic microorganisms can also methylate arsenic in arsenic-contaminated soils *in vitro.*^66^ However, there is still no direct evidence that the microbial metabolism of arsenic is of toxicological significance for the host.

Bismuth is widely used in pharmaceuticals, cosmetics, catalysis, industrial pigments, alloys and ceramic additives. Faeces samples from human volunteers and isolated gut segments from mice can transform bismuth into its toxic volatile derivative trimethylbismuth *ex vivo* during anaerobic incubation, whereas gut segments of GF mice are unable to perform this conversion.^67^ Remarkably, the production rates of volatile derivatives of other elements such as arsenic, antimony, tin and lead, were also higher, demonstrating synergetic interactions in metalloid transformation reactions. As volatile derivatives are usually more toxic than their inorganic precursors, these bacteria-mediated transformations may have a significant impact on the host, but *in vivo* evidence is warranted.

#### Benzene derivatives

Benzene derivatives, such as nitrobenzene, are the starting materials for the synthesis of numerous aromatic nitro compounds and aromatic amines. Exposure of experimental animals to nitrobenzene induces methemoglobinemia, impacts the nervous system, causes liver necrosis and degeneration of the seminiferous epithelium.^68^ However, no methaemoglobin is detected in GF or antibiotic-treated rats upon exposure to nitrobenzene.^69,70^ *Ex vivo* metabolism studies with isolated caecal content demonstrated that the GI microbiota sequentially reduced nitrobenzene to the potentially toxic intermediates nitrosobenzene, phenylhydroxylamine and aniline.^70^ Antibiotic treatment drastically diminishes excretion of reductive metabolites, offering protection against nitrobenzene-induced methaemoglobinaemia.^70^ Thus, the GI microbiota is clearly a major determinant of the metabolism and toxicity of nitrobenzene.

To the contrary, GI bacteria seem to protect against 1,3-dinitrobenzene toxicity, which causes methaemoglobinemia in humans and laboratory animals. The administration of a single oral dose of 1,3-dinitrobenzene caused central nervous system toxicity (ataxia) in GF rats but not in conventional rats, whereas repeated oral dosing with 1,3-dinitrobenzene was required to cause ataxia in conventional rats.^71^ Considerable differences in the uptake, tissue distribution and excretion of 1,3-dinitrobenzene were observed between GF and conventional rats^71^ but it cannot be concluded whether these differences are due to direct metabolism by GI bacteria or to physiological differences such as variations in the absorptive properties of the gut wall that are altered in GF animals.

#### Azo dyes

Azo dyes are a group of compounds containing one or more R_1_-N=N-R_2_ bonds. At least 3,000 azo dyes are used in the textile, paper, food, cosmetics and pharmaceutical industries. Five azo dyes are used as food colourants: Citrus Red No. 2, Allura Red, Tartrazine, Sunset Yellow and Orange B. These commonly used azo dyes have no adverse cytotoxic, mutagenic or carcinogenic effects. Other azo dyes, the Sudan dyes, are used in plastics, printing inks, waxes, leather, fabrics and floor polish. Although Sudan dyes are banned for food usage in most countries, they are illegally used to maintain the colour in food products. Human exposure to Sudan dyes and Para Red occurs through ingestion, inhalation or skin contact. Several studies have provided unequivocal evidence that azoreductases from the GI microbiota catalyse cleavage of the azo bond of numerous azo dyes to produce potentially carcinogenic aromatic amines.^72^ For example, human faecal suspensions can metabolise Sudan I and II and Para Red to aniline, 2,4-dimethylaniline, *o*-toluidine and 4-nitroaniline.^73^ *In vivo*, azobenzene is reduced to aniline (aminobenzene) in conventional rats but not in GF rats.^74^ These bacteria-dependant metabolites are classified as carcinogen (*o*-toluidine) or potential carcinogen (aniline) for humans.

#### Melamine

Melamine has received intense media attention as the cause of renal failure and death in both animals and children in China in 2008, following its addition to pet food and infant formula to boost apparent protein content. This renal damage is believed to result from kidney stones formed from melamine and uric acid, or from melamine and cyanuric acid. Zheng *et al.* demonstrated that melanine toxicity was significantly attenuated in antibiotic-treated rats and that melamine is converted to cyanuric acid *in vitro* by bacteria cultured from rat faeces. They subsequently identified a species of *Klebsiella* in faeces samples that was able to generate cyanuric acid from melamine. Finally, rats colonised with *Klebsiella terrigena* display exacerbated melamine-induced nephrotoxicity.^75^ The renal toxicity of melamine is thus mediated by the GI microbiota and may depend on the exact composition and metabolic activities of the GI microbiota.

#### Artificial sweeteners

Artificial sweeteners were introduced into the human diet more than a century ago to decrease caloric intake and are now widely found in commonly consumed foods such as diet soft drinks and food. The impact of artificial sweetener consumption on health is a matter of intense debate. Some studies have shown benefits of their consumption,^76^ whereas others have suggested associations with increased risk of type 2 diabetes.^77^ Cyclamate is one of the most widely used artificial sweeteners in Europe. It is metabolised into cyclohexamine, which is thought to be responsible for the carcinogenic effect of cyclamate,^78^ an effect that resulted in the banning of cyclamate in the UK and the US, although this toxicity is still controvertial. Interestingly, cyclamate metabolism is inducible: cyclohexamine was detected as the main urinary metabolite of ^14^C-cyclamate in rats, rabbits, guinea pigs and humans, in individuals chronically consuming cyclamate before administration of the radiolabelled dose.^79^ Evidence implicating the GI microbiota as the site of cyclamate metabolism are numerous: cyclamate was found to be converted to cyclohexamine *in vitro* by the contents of the lower gut but not by the tissues of rats pretreated with cyclamate;^80^ rats given cyclamate in drinking water for several months became ‘converters’, excreting cyclohexamine, but this ability to convert cyclamate was lost when antibiotics were added to water.^81^ Many publications have extended these observations to humans,^82^ providing evidence that the GI microbiota is the sole site of cyclamate metabolism.

## Environmental chemicals can affect the composition and/or the metabolic activity of the GI microbiota

The composition, diversity and enzymatic capacities of the gut microbiota are readily affected by various environmental factors, such as the host’s lifestyle, diet and use of antibiotics.^83,84^ Several environmental chemicals have also been shown to inhibit GI bacterial growth or to induce dysbiosis *in vitro* and *in vivo.* As discussed previously, dysbiosis of the gut microbiota has been linked to a series of intestinal and systemic diseases.^85^ Interestingly, most of these disorders have also been linked to exposure to environmental chemicals. For example, there has been a tremendous increase in the prevalence of allergic diseases such as asthma and food allergies in the last few decades. Recent evidence suggests that both alterations of microbial colonisation during the perinatal period and early-life exposure to environmental chemicals may promote dysregulated immune responses.^86–88^ It therefore seems plausible that exposure to chemicals may affect the normal colonisation of the gut by bacteria, with effects on host physiology later in life. Therefore, chemical-induced perturbations of the composition of the GI microbiota may constitute an underestimated mechanism by which they interfere with human health (Figure 1c).

Moreover, there is a growing consensus that a healthy microbiome might not be defined as an idealised assembly of specific microbe populations, but that the microbes community should be able to perform a set of metabolic functions together with its host, although this set of metabolic functions is still to be defined.^85^ This is of particular importance because some xenobiotics might impact the GI microbiota physiology without inducing dysbiosis: indeed, when fresh human faecal samples were incubated with antibiotics or with host-targeted drugs, all host-targeted drugs resulted in significant alterations of microbial gene expression, including genes involved in xenobiotic metabolism, despite minimal short-term effects on microbial community structure.^89^ Such interactions may affect the metabolism, toxicity, and risk assessment of many environmental compounds that become toxic upon microbiome-mediated metabolism.

We present below the environmental chemicals that have been shown to affect the composition of the GI microbiota and those interfering with the metabolic activity of gut microbes. These compounds are also described in Table 1 and in more details in Supplementary Table 2.

### Pesticides

Glyphosate is the active component of Roundup (Montsanto, St Louis, MO, USA), the most widely used herbicide worldwide. It has been shown that the growth of *Enterococcus faecalis* bacteria isolated from cattle and horse faeces is inhibited by the lowest concentrations of glyphosate and the herbicide formulation tested.^90^ Further studies demonstrated that sensitivity to glyphosate is dependent on the bacterial strain. In particular, in poultry, pathogenic bacteria such as *Salmonella enteritidis, Salmonella gallinarum, Salmonella typhimurium, Clostridium perfringens* and *Clostridium botulinum* are highly resistant to glyphosate, whereas beneficial bacteria such as *Enterococcus faecalis, Enterococcus faecium, Bacillus badius, Bifidobacterium adolescentis* and *Lactobacillus sp.* are moderately to highly susceptible.^91^ If it were demonstrated that glyphosate induced similar dysbiosis in mammals, then this would be of toxicological relevance because it would lead to a reduction of potentially beneficial bacteria in the GI tract of the host.

Chlorpyrifos is an organophosphate insecticide commonly used to treat fruit and vegetable crops and vineyards. The effect of chronic exposure to chlorpyrifos has been investigated in an *in vitro* simulator of the human GI tract inoculated with faeces from healthy humans, and *in vivo* in rats exposed from *in utero* until 60 days old.^92^ Exposure to chlorpyrifos induced dysbiosis of the microbial community that was associated with proliferation of *Bacteroides sp*. and decreased levels of *Lactobacillus sp.* and *Bifidobacterium sp*. To the best of our knowledge, this is so far, the only study that has investigated the effect of perinatal exposure to low doses of environmental chemicals on GI colonisation.

### Metals

In mice supplied with cadmium-containing drinking water, a sharp decrease in the populations of all microbial species was observed.^93^ Breton *et al.* also reported minor but specific changes in the composition of the colonic microbiota at the family and genus levels, following chronic treatment with cadmium and lead in mice. Heavy metal-consuming animals had smaller numbers of *Lachnospiraceae* and larger numbers of *Lactobacillaceae* and *Erysipelotrichaceacae* than control animals.^94^ Exposures to these heavy metals were subtoxic and not associated with hepatotoxicity, changes in behaviour, organ and body weights, food intake, stool consistency or gut motility. However, low number of *Lachnospiraceae* has been previously linked to intestinal inflammation and suggested as a predisposition to colitis.^95^ Similarly, 4-week arsenic treatment induced a significant decrease in mouse faecal populations of Firmicutes, but not in Bacteroidetes. These taxonomic changes were associated with an alteration of the metabolic activity of the gut microbiome as demonstrated by the variation of a number of microbial co-metabolites, such as indole derivatives, observed in urine or faeces of exposed animals.^96^

### Other persistent organic pollutants

Persistent organic pollutants are highly resistant to environmental and biological degradation and therefore persist in the environment and accumulate in the food chain. Persistent organic pollutants are ligands of the aryl hydrocarbon receptor (AhR), which mediates the toxic effects of these compounds. Zhang *et al.*^97^ recently (2015) demonstrated the key role of AhR activation in promoting the toxic effect of 2,3,7,8-tetrachlorodibenzofuran (TCDF) on gut bacteria using AhR^−/−^ mice fed a diet enriched with TCDF (0.6 μg/kg per day) for 5 days. TCDF modulated the gut microbial balance by strongly depleting segmented filamentous bacteria in the ileum of AhR^+/+^ but not in AhR^−/−^ mice. TCDF also decreased the Firmicutes/Bacteroidetes ratio in an AhR-dependant manner. These changes were accompanied by strong metabolic differences in the faeces, caecal content, liver and gut tissues between TCDF-treated and control AhR^+/+^ mice, whereas TCDF had no effect on the metabolic profiles of AhR^−/−^ mice. In particular, TCDF decreased glucose and oligosaccharide concentrations and increased the levels of short-chain fatty acids (butyrate and propionate) in faeces and caecal contents, consistent with an activation of bacterial fermentation. These findings demonstrate that exposure to persistent organic pollutants has potentially a major impact on the host-microbiota metabolic axis, through the activation of AhR signalling. The precise mechanism of TCDF toxicity to gut bacteria remains to be elucidated. Moreover, this study used high doses of TCDF, so it remains to be determined whether similar changes are observed at environmental levels of exposure.

### Artificial sweeteners

As previously discussed, cyclamate metabolism is inducible, but the increase in sulfamase activity does not seem to be associated with any significant taxonomic changes.^98,99^ However, a slight increase in *Clostridium* levels has been reported following the chronic administration of cyclamate in rats.^98^ Similarly, acesulfame potassium does not seem to possess significant anti-bacterial nor bacterial growth-promoting effects.^100^ Nevertheless, these studies were performed using culture-based methods of bacterial detection and it may be necessary to re-assess the impact of cyclamate and acesulfame potassium on the gut microbial ecosystem using modern sequencing technologies. On the contrary, there is growing evidence that aspartame, sucralose and saccharin induce significant dysbiosis in animals and humans and that this dysbiosis is responsible for deleterious metabolic effects in the host. In rats treated with Splenda (Heartland Consumer Products, Carmel, IN, USA) (the commercial formulation of sucralose, containing 1.1% w/w sucralose plus maltodextrin and glucose), faecal counts of total anaerobes increased, whereas the numbers of bifidobacteria, lactobacilli, *Bacteroides*, clostridia and total aerobic bacteria decreased.^101^ This represents a reduction in the levels of beneficial bacteria to the host. Similarly, in rats fed low doses of aspartame in drinking water (5–7 mg/kg per day), an increase in total bacteria and in *Enterobacteriaceae* and *Clostridium leptum* levels was observed.^102^ Aspartame-consuming rats also had higher serum propionate levels and higher fasting glucose levels. The authors suggested that aspartame directly altered the gut microbiota, favouring propionate production, and hypothesised that the end result might be an increase in hepatic gluconeogenesis. This hypothesis is also supported by the recent work of Suez *et al.*,^103^ who treated mice by adding saccharin, sucralose or aspartame (5% sweetener+95% glucose) to drinking water and observed that artificial sweetener-consuming mice developed glucose intolerance, whereas no such intolerance was observed in mice drinking water or glucose alone. This artificial sweetener-induced glucose intolerance was mediated by changes to the commensal microbiota, with contributions from diverse bacterial taxa, because treatment with broad spectrum antibiotics protected animals against glucose intolerance phenotype. Using saccharin as a prototype sweetener at a dose corresponding to the ADI for humans (5 mg/kg per day), the authors further characterised the faecal microbiota composition of saccharin-consuming mice and demonstrated that saccharin induced strong dysbiosis, with an increase in the number of bacteria from the *Bacteroides* genus and the *Clostridiales* order. In stool samples from saccharin-consuming mice, glycan-degradation pathways were over-represented. Fermentation of glycans leads to the production of various compounds, including short-chain fatty acids. Consistent with this finding, faeces from saccharin-consuming mice had high propionate and acetate levels. Finally, in a small intervention study in humans, most of the volunteers (4/7) developed a significantly poorer glycemic response after 5–7 days of saccharin consumption. The microbiota of these four responders changed markedly during the intervention period and clustered differently from the one of the three non-responders, in whom blood glucose levels were not affected post-saccharin treatment.^103^ These results suggest that human individuals feature a personalised response to artificial sweeteners, possibly stemming from their differences in microbiota composition and function.

### Conclusions and perspectives

This review presents various mechanisms underlying bidirectional interactions between environmental pollutants and GI microbiota. The GI bacteria have broad enzymatic capacities and can metabolise environmental chemicals from various chemical families, either increasing or decreasing their toxicity to the mammalian host. Conversely, environmental chemicals may also affect the composition and/or optimal function of the GI microbiota, with potential effects on the health of the host. Overall, we estimate that the GI microbiota represent a major player in the toxicity of environmental contaminants. Nevertheless, there remain many challenges to overcome in order to establish the level of risk associated with environmental pollutants in interaction with gut bacteria. The mechanisms of interaction between gut bacteria and endogenous enzymes involved in drug detoxification pathways must be identified. This is necessary to evaluate the impact of GI microbiota variations on endogenous xenobiotic metabolism and overall susceptibility to environmental chemicals. Another challenge is to assess the impact of long-term exposure to low-dose environmental chemicals on the gut microbial ecosystem. To the best of our knowledge, no study has investigated this question yet. Importantly, the relevance of these microbiome-xenobiotics interactions on human health needs to be assessed. This is particularly relevant to metabolic disorders that have already been associated with modulations of the gut microbiome. In this context, exposure to environmental chemicals during the perinatal period and early childhood may have a critical impact on the development of the GI microbiota and related microbiota-dependent host physiological functions, such as immune-system development. The consequences of early exposure to pollutants must therefore be investigated as a new factor potentially affecting the development of health disorders later in life.

## Figures and Tables

**Table 1:** Human exposure to pollutants and their interaction with the GI microbiota

*Chemical*	*Source*	*Human exposure*	*Metabolism by microbiota*	*Effect on microbiota*	*References*
PAHs	Air and food pollutants resulting from incomplete combustion of fossil fuel, tobacco	Mean total intake of 3.12 mg per day (97% through food, 1.6% air, 0.2% water, 0.4% soil)	*In vitro*: hydroxylation; *in vivo*: deconjugation of liver metabolites, involved in the formation of CH_3_S-metabolites		32,104,105
Nitro-PAHs	Air and food pollutants, derivatives of PAHs	Diesel exhaust identified as main source of exposure. 2NF: range from 0 to 92 ng/m^3^	Reduction to amine metabolites		36,106
Nitrotoluenes	Intermediates in the manufacture of dyes, chemicals, explosives	Mainly occupational. 2-nitrotoluene: 0.35–0.7 mg/m^3^ through air; 420 mg per day through skin	Reduction to amine metabolite and hydolysis of glucuronide conjugates		39,40,107
Pesticides	Pollutants in air and food	Chlorpyrifos: mainly through diet 0.01 to 0.14 μg/kg bw per day; DDT: through diet 0.29 μg/kg bw per day	Dechlorination of organochlorides. Deconjugation of propachlor *in vivo*	Perinatal exposure to chlorpyrifos (1 mg/kg bw per day) induced dysbiosis at adulthood (rat)	45,92,108
PCBs	Industrial chemicals now prohibited but persistent in water sediments and soils	Mainly through diet DL-PCBs: 0.29 pg TEQ WHO_98_/kg bw per day; NDL-PCBs: 2.71 ng/kg bw per day	Bacterial C–S-lyase plays an important role in formation of methyl sulfone (MeSO_2_)-metabolites *in vivo*	Mixture of PCBs (150 μM/kg for 2 days) decreased the abundance of many bacteria (mainly Proteobacteria)	55,56,109,110
Metals	Ubiquitous environmental contaminants	Mainly through diet: arsenic 0.78 μg/kg bw per day; lead 0.2 μg/kg bw per day; cadmium 0.16 μg/kg bw per day	Involved in demethylation of mercury, methylation of arsenic and bismuth	Cadmium (20–50 mg/kg bw/d for 45 days); lead (100 or 500 mg/l) or arsenic (10 p.p.m. for 4 weeks) induced dysbiosis (mouse)	93,94,96,109
Azo dyes	Food colourants	Mainly through diet	Azoreduction of the azo bound to produce aromatic amines		74,111
Melamine	Widely used in plastics, illegal food contaminant	TDI: 0.2 mg/kg bw (EU)	Metabolised to cyanuric acid		75
Artificial sweeteners	Food additives	ADI (FDA, US): Aspartame: 50 mg/kg bw; saccharin: 15 mg/kg bw	Cyclamate metabolised to cyclohexamine	Aspartame (5–7 mg/kg/d), sucralose and saccharin (5 mg/kg per day) induce dysbiosis in animals with potential deleterious metabolic effect for the host (mouse and human)	80,98,102,103
Other POPs (e.g., PCDFs)	Pollutants formed during industrial processes	Mainly through diet: PCDD/Fs 0.176 pg TEQ WHO_98_/kg bw per day		2,3,7,8 TCDF (24 μg/kg) induced dysbiosis and affected the faecal metabolic profiles (mouse)	97,109

Abbreviations: ADI, acceptable daily intake; DL-PCBs, dioxin-like PCBs; EU, European Union; FDA, Food and Drug Administration; NDL-PCBs, Non-dioxin-like PCBs; PCBs, polychlorobiphenyls; PAHs, polycyclic aromatic hydrocarbons; POPs, persistent organic pollutants; TEQ, toxic equivalency; TDI, tolerable daily intake.
