# The gut microbiota: a major player in the toxicity of environmental pollutants?

**DOI:** 10.1038/npjbiofilms.2017.1

**Published:** 2017-06-22

**Authors:** Sandrine P Claus, Hervé Guillou, Sandrine Ellero-Simatos

**Correction to:** *npj Biofilms and Microbiomes* (2016) **2**, 16003; doi: http://dx.doi.org/10.1038/npjbiofilms.2016.3; published online 4 May 2016

In the Supplementary Information File originally published with this Article, the tracked changes were visible within the document. This error has now been corrected in the Supplementary Information that now accompanies the Article.

A correction has been published and is appended to both the HTML and PDF versions of this paper. The error has been fixed in the paper.

