# Supplementary material for: The gut microbiota: a major player in the toxicity of environmental pollutants?
Source: NPJ Biofilms Microbiomes. 2016 May 4;2:16003–. doi: 10.1038/npjbiofilms.2016.3 (PMC5515271; doi:10.1038/npjbiofilms.2016.3)
Supplement: Supplementary Information [file npjbiofilms20163-s1.pdf]

The Gut Microbiota: A Major Player in the Toxicity of Environmental Pollutants?

Sandrine P. Claus, Hervé Guillou, Sandrine Ellero-Simatos

Supplemental Material

Table of contents:

Supplemental Table 1: Environmental chemicals metabolized by the GI microbiota…...Page 2

Supplemental Table 2: Environmental chemicals that interfere with the composition or the activity of the GI microbiota……………………………………………………………..…Page 7

Supplemental Table 1: Environmental chemicals metabolized by the GI microbiota

Compound			Experimental protocol			
**Class**	**Name**	**Use**	**Enzyme sources**	**Parameters evaluated**	**Results**	**Reference**
PAHs	Benzo(a)-pyrene	Found in diesel engine emission, cooked meat	SHIME inoculated with human feces	Metabolism, yeast estrogen bioassay	Colonic digests became estrogenic. 7-OH benzo(a)pyrene detected in colonic digests.	(Van de Wiele et al. 2005)
	Naphthalene	Insecticide, used as precursor for plasticizer synthesis	SHIME inoculated with human feces	Metabolism, yeast estrogen bioassay	Colonic digests became estrogenic.	(Van de Wiele et al. 2005)
			Bile cannulated rats, GF vs. conv rats	Metabolism	Enterohepatic circulation of naphtalene glutathion conjugates. Microbiota involved in the production of CH_3S-metabolites and the naphthols.	(Bakke et al. 1985)
	Phenanthrene	Found in cigarette smoke	SHIME inoculated with human feces	Metabolism, yeast estrogen bioassay	Colonic digests became estrogenic.	(Van de Wiele et al. 2005)
	Pyrene	Used as dye and dye precursor	SHIME inoculated with human feces	Metabolism, yeast estrogen bioassay	Colonic digests became estrogenic. 1-OH pyrene detected in colonic digests.	(Van de Wiele et al. 2005)
Nitro-PAHs	2-nitrofluorene	By-product of combustion	GF and conv rats treated orally	Metabolism, Ames test	7-, 5-, 3-, 1-OH acetylaminofluorene major metabolites in conv rats, while only OH-NF in GF rats. Mutagenicity increased in urine and feces of GF rats.	(Möller et al. 1988)
			GF and conv rats treated orally	DNA adduct formation in liver, kidney, lung, heart	More DNA adducts in conv animals in all tissues studied.	(Möller et al. 1994)
	6-nitrobenzo(a)-pyrene	Found in engine emission	Fecal suspensions (human and rat)	Metabolism	Reduced to 6-aminobenzo(a)pyrene	(Cerniglia et al. 1984)
			Human fecal suspensions (anaerobic)	Metabolism, Ames test	Reduced to 6-nitrosobenzo(a)pyrene and 6-aminobenzo(a)pyrene. 6-nitrosobenzo[a]pyrene exhibited strong direct-acting mutagenicity.	(Fu et al. 1988)
	3-nitrobenzo(a)-pyrene	Found in engine emission	Rat fecal suspensions (anaerobic)	Metabolism	Reduced as 3-aminobenzo(a)pyrene	(Richardson et al. 1988)
	6-nitrochrysene	Found in ambient air	SHIME inoculated with human feces. Fecal suspensions (human, mouse, rat)	Metabolism	Reduced to 6-aminochrysene	(Manning et al. 1988)

Compound			Experimental protocol			
Class	Name	Use	Enzyme sources	Parameters evaluated	Results	Reference
Nitro-PAHs	1-nitropyrene (1-NP)	By-product of combustion in diesel engine	Suspension of intestinal content (rats)	Metabolism	Metabolized to 1-aminopyrene	(Howard et al. 1983)
			Semi continuous culture system	Ames test	Metabolites were non mutagenic	(Manning et al. 1986)
			Fecal suspension (human, rat, mouse)	Ames test	Mutagenicity was reduced	(King et al. 1990)
			Fecal suspension (human)	Metabolism, Ames test	*C leptum, C paraputrificum, C clostridiiforme*, another *Clostridium* sp. and a *Eubacterium* sp. were able to metabolize 1-NP. Metabolites were not mutagenic.	(Rafil et al. 1991)
			GF and conv Fisher rats treated orally	Metabolism	Reduced to 1-aminopyrene in conv but not in GF rats.	(El-Bayoumy et al. 1983)
			GF and conv F344 rats treated orally	Metabolism	1-aminopyrene, 1-amino-6-hydroxypyrene, 1-amino-8-hydroxypyrene detected in urine and feces of conv rats but not in GF rats	(El-Bayoumy et al. 1983)
			Conv and GF Wistar rats	Radioactivity concentrations, covalent binding, Ames test, metabolism	Covalent binding of 1-NP metabolites greater in liver and kidney from conv rats, and in GI tract from GF rats. Fecal extracts and urine from conv rats more mutagenic in presence of an S9 mix, while those from GF rats more mutagenic in the absence of S9. Glucuronide conjugates major metabolites in urine of conv rats.	(Kinouchi et al. 1986)
			Conv and GF AGUS rats, i.p. injection.	Metabolism, Ames test	Metabolized to 6-hydroxy-1-acetamidopyrene in conv rats. Urine from conv rats is more mutagenic than that from GF rats.	(Ball et al. 1991)
			Mice +/- antibiotics gavaged with glutathione conjugates of 1-NP oxides	Ames test and DNA adduct formation	Specific DNA adducts were detected in the saline-treated group but not in the antibiotics-treated group	(Kinouchi et al. 1992)
			Beta-lyase from *Peptostreptococcus magnus*	Ames test	Cysteine conjugates of 1-NP oxides are changed to a more genotoxic form by b-lyase-mediated deconjugation	(Kataoka et al. 1995)
Dinitro-toluenes (DNT)	2-nitrotoluene	Used in the production of polyurethane foams, automobile air bags, dyes and explosives	GF and conv F344 rats treated orally	DNA repair	No induction of DNA repair in GF animals whereas DNA repair was induced in conv males.	(Doolittle et al. 1983)
	2,4-dinitrotoluene		GF and conv F344 rats treated orally	Metabolism & hepatic covalent binding	4-N-acetyl-2-nitrobenzoic acid and 2-amino-4-nitrobenzoic acid detected in conv but not GF animals. Hepatic covalent binding was decreased in GF rats.	(Rickert et al. 1981)
			Caecal suspension (mouse, rat). Fecal suspension (human)	Metabolism	Reduced to 2-amino-4-nitrotoluene, 2-nitro-4-aminotoluene and 2,4-diaminotoluene	(Guest et al. 1982)
	2,6-dinitrotoluene		GF and ex-GF rats treated orally	Autoradio-graphic assay	Absence of unscheduled DNA synthesis in GF rats	(Mirsalis et al. 1982)

Compound			Experimental protocol			
**Class**	**Name**	**Use**	**Enzyme sources**	**Parameters evaluated**	**Results**	**Reference**
Pesticides	DDT	Organo-chlorine pesticide	*Eubacterium limosum* isolated from human GI microbiota	Metabolism	Metabolized to 1,1-dichloro-2,2-bis(p-chlorophenyl)ethane (DDD)	(Yim et al. 2008)
			Rats injected by stomach tube or ip; cultures of *E. Coli* or *A. aerogenes* isolated from rat feces	Metabolism	DDD detected in rats receiving DDT by stomach tube not ny ip. DDD detected in bacterial cultures.	(Mendel and Walton 1966)
	Propachlor	Acetamide herbicide	Bile cannulated rats, GF v. conv rats	Metabolism	Glutathion conjugate, cysteine conjugate, mercapturate and mercapturate sulfoxide found in bile. Water soluble metabolites of propachlor only in feces of GF rats.	(Gustafsson et al. 1981)
			GF vs. conv rats	Metabolism	N-acetyl-cysteine and cysteine conjugates main metabolites in GF rats. In conv rats, 11 metabolites detected in urine, 6 of which were 2-methylsulfonylacetanilides not detected in GF rats.	(Bakke et al. 1980)
	Methoxychlor	Organo-chlorine insecticide	*Eubacterium limosum* isolated from human feces	Metabolism	Metabolized to methoxydichlor	(Yim et al. 2008)
	Lindane	Organo-chlorine pesticide	Cecal content (rat)	Metabolism	Dechlorinated to 3,4,5,6-Tetrachlorocyclohex-1-ene	(Stein et al. 1980)
Metals	Methyl-mercury	Environmental contaminants of aqueous ecosystems	Small intestine and caecum content from rats	Metabolism	Metabolized to a volatile product	(Rowland et al. 1978)
			GF vs conv mice	Excretion, accumulation	Mercury excretion was lower in feces from GF mice. Mercury retention in organs was higher in GF mice.	(Nakamura et al. 1977)
			Antibiotics vs conv mice	Excretion, accumulation	Mercury excretion was lower in antibiotics-treated mice.	(Seko et al. 1981)
			Antibiotics vs conv rats	Excretion, accumulation	Mercury excretion was lower in feces from antibiotics-treated rats. Mercury retention in organs was higher in antibiotics-treated rats.	(Rowland et al. 1980)
	Arsenic (As)	Ubiquitous environmental contaminant	Feacal suspensions (human); SHIME	Metabolism	Both inorganic As and soil-contaminated As were metabolized into methylated arsenicals and thioarsenicals	(Van de Wiele et al. 2010)
			WT vs. MDR2-deficient rats	Metabolism	Generation of thioarsenicals dependant of enterohepatic ciculation. Arsenicals excreted from bile transformed into MMMTA(V) and DMMTA(V)	(Bu et al. 2011)
	Bismuth	Used in pharma, cosmetics	Feces samples (human); isolated gut segments of conv and GF mice	Metabolism	Human feces and isolated gut segments of conv mice produced trimethylbismuth [$(CH_3)_3Bi$], gut segments of GF mice did not.	(Michalke et al. 2008)

Compound			Experimental protocol			
**Class**	**Name**	**Use**	**Enzyme sources**	**Parameters evaluated**	**Results**	**Reference**
PCBs	2,4',5-trichloro-biphenyl	Were widely used as dielectric and coolant fluids	Bile cannulated rats, GF v. conv rats	Accumulation	Fat from conv rats contained 3-15 times more radioactivity than GF. 95% of radioactivity detected in bile of conv rats.	(Gustafsson et al. 1981)
			GF vs. conv mice	Accumulation, Metabolism	4-MeSO_2-triCB higher in lung, kidneys and liver of conv mice	(Brandt et al. 1982)
Benzene derivatives	Nitrobenzene	Used in the production of aniline, which is a precursor to rubber chemicals, pesticides, dyes, explosives and pharma	GF, antibiotic treated or ex-GF rats treated ip	Methemoglobon in determination	No methemoglobin in GF or antibiotic treated rats.	(Reddy et al. 1976)
			Cecal suspensions; Conv and antibiotic treated rats	Metabolism	Reduced to nitrosobenzene, phenylhydroxylamine and alinine *in vitro*. In antibiotic treated rats, urinary excretion of p-hydroxyacetanilide reduced.	(Levin and Dent 1982)
	1,3 dinitrobenzene	Used in the manufacture of explosives	Conv, GF and ex-GF F-344 rats treated ip	Methemoglobon in determination	GF rats produced less methemoglobin than conv rats. Ataxia in GF but not in conv or ex-GF rats. Higher concentrations of 1,3-dinitrobenzene radioactivity in brain, liver and plasma from GF.	(Philbert et al. 1987)
Azo dyes	Azobenzene	Colorants in food products	GF vs conv rats	Metabolism	Reduced to aniline only in conv. rats.	(Macholz et al. 1985)
	Sudan I, II, III, IV and ParaRed		Human fecal suspensions (anaerobic)	Metabolism	Metabolized to aniline, 2,4-dimethylaniline, o-toluidine, and 4-nitroaniline.	(Xu et al. 2007)
	Sudan I, II, III, IV		Human intestinal bacteria (isolated strains)	Metabolism	*Bifidobacterium infantis*, *Clostridium indolis*, *Enterococcus faecalis*, *Lactobacillus rhamnosus* and *Ruminococcus obeum* able to reduce the 4 dyes. *Escherichia coli* and *Peptostreptococcus magnu*s not able to reduce any of the 4 dyes.	(Xu et al. 2010)
Miscella-neous	Melamine	Plasticizer, food contaminant	Fecal suspensions (rats). Rats treated with antibiotics	Metabolism, Kidney histology	*Klebsiella* converted melamine to cyanuric acid. Antibiotic treatment reduced melamine-induced kidney damage. Colonisation by *Klebsiella terrigena* exacerbated melamine-induced nephrotoxicity.	(Zheng et al. 2013)
	Haloacetic acids	By-products of drinking water disinfection	Rat cecal suspensions	Salmonella microsuspensio n bioassay	Presence of microflora did not alter mutagenicity.	(Nelson et al. 2001)
	Butyl paraben	Anti-microbial preservatives	Fecalase mixture from human feces	Cytotoxicity and apoptosis	Metabolized to paraben, thereby reducing cytotoxicity and apoptosis in HepG2 cells.	(Khanal et al. 2012)

Compound			Experimental protocol			
Class	Name	Use	Enzyme sources	Parameters evaluated	Results	Reference
Artificial sweeteners	Cyclamate	Used as sugar substitute in a variety of food product	^{14}C-labelled cyclamate incubated with preparations of liver, kidney, spleen, blood and lower gut contents of cyclamate-preteated or of control rats	Metabolism	Cyclamate converted to cyclohexamine by the lower gut content of cyclamate pretreated animals	(Renwick and Williams 1969)
			^{14}C-labelled cyclamate administered to guinea pigs, rabbits, rats and humans	Metabolism	When given orally, cyclamate is excreted unchanged. When the same animals are placed on a cyclamate-containing diet, cyclamate is converted to cyclohexamine.	(Renwick and Williams 1972)
			Rats given cyclamate and antibiotics in drinking water	Metabolism	Rats given cyclamate for 6-15 months converted cyclamate to cyclohexamine. Co-treatment with antibiotics suppressed the ability to convert cyclamate to cyclohexamine	(Bickel et al. 1974)
			Cecum and colon contents, feces of cyclamate-pre treated rats, rabbits, guinea pigs and humans	Metabolism	Cyclamate was converted into cyclohexylamine when incubated with rats, rabbits and 1/3 human samples, not guinea pigs. The gut organisms converting cyclamate into cyclohexylamine are clostridia in rat, enterobacteria in rabbit and enterococci in man.	(Drasar et al. 1972)
	Saccharin	Sugar substitute	^{14}C-labelled saccharin administered orally to rats and rabbits on a normal diet or rats and rabbits given a saccharin-enriched diet for months.	Metabolism	No metabolite detected	(Ball et al. 1977)
			Fecal homogenates from rats kept on a saccharin diet for 2 years	Metabolism	No metabolite detected	(Ball et al. 1977)
			Several animal species and humans + human intestinal strains	Metabolism	No metabolite detected	(Rymon Lipinski 1985)

Supplemental Table 2: Environmental chemicals that interfere with the composition or the activity of the GI microbiota

Compound			Experimental protocol			
**Class**	**Name**	**Use**	**Exposure protocole**	**Parameters evaluated**	**Results**	**Reference**
Pesticides	Glyphosate	Most widely used herbicide in the world, active component of Round Up	*E. faecalis* isolated from feces of cattle and horse incubated with glyphosat or roundup (0-10 mg/ml) for 8 h	Bacterial growth	0.1 mg/ml Glyphosate and round up inhibited *E. Faecalis* growth	(Krüger et al. 2013)
			Various bacterial strains isolated from chicken	Bacterial growth	Highly pathogenic bacteria (*S. Entritidis, S. Gallinarum*…) highly resistant to glyphosate (Minimal inhibitory concentrations 5 mg/ml), whereas beneficial bacteria (*E. faecalis*…) moderate to highly susceptible (MIC 0.07-0.3 μg/ml)	(Shehata et al. 2013)
	Chlorpyrifos	Organophosphate insecticide	SHIME inoculated with human feces	Bacterial count	Slight increase in the total aerobic and anaerobic counts; strong increase over time in the numbers of *Enterococcus spp*. and a moderate increase in the numbers of *Bacteroides spp*. Decrease in *Lactobacillus sp*. and *Bifidobacterium sp* counts	(Joly et al. 2013)
			Rats gavaged with 1 mg/kg/day from gestation to PND60	Bacterial count	The total anaerobic counts did not differ significantly. No difference in *Enterococcus spp*. or *Bacteroides spp*., slight increase in the ileum and the colon but not in the cecum. *Lactobacillus sp*. counts significantly lower in ileum, cecum and colon. Decrease in *Bifidobacterium spp*. count in the colon	
Metals	Cadmium	Ubiquitous environmental contaminants	Mice exposed to 23 to 50 mg/kg in drinking water for 45 days	Bacterial count	Sharp decrease in the population of all intestinal bacteria	(Fazeli et al. 2011)
			Mice exposed to 20 or 100 ppm in drinking water for 8 weeks	16S rRNA gene sequencing	Decreased *Lachnospiraceae* and increased *Lactobacillaceae* and *Erysipelotrichaceacae* (mainly due to changes in *Turicibacter sp*)	(Breton et al. 2013)
	Lead		Mice exposed to 100 or 500 ppm in drinking water for 8 weeks	16S rRNA gene sequencing	Decreased *Lachnospiraceae* and increased *Lactobacillaceae* and *Erysipelotrichaceacae* (mainly due to changes in *Turicibacter sp*)	(Breton et al. 2013)
	Arsenic		Mice exposed to 10 ppm inorganic arsenic in drinking water for 4 weeks	16S rRNA gene sequencing	No changes in Bacteroides. Significant changes in 6 families of Firmicutes.	(Lu et al. 2014)

Compound			Experimental protocol			
**Class**	**Name**	**Use**	**Exposure protocole**	**Parameters evaluated**	**Results**	**Reference**
Artificial sweeteners	Aspartame	Sugar substitutes	Rats fed chow or HF diet +/- aspartame (5-7 mg/kg/day) in drinking water for 8 weeks	qRT-PCR of 16S rRNA	Aspartame increased total fecal bacteria and the abundance of *Enterobacteriaceae* and *Clostridium leptum*. Aspartame decreased the HF-induced increase in the Firmicutes/Bacteroidetes ratio.	(Palmnäs et al. 2014)
				Metabolomics	Increased serum levels of propionate	
	Cyclamate		Rats kept on a cyclamate diet for 4 months	Bacterial count	Significant increase in clostridia counts in feces.	(Drasar et al. 1972)
			Rat fecal microbiota maintained under continuous flow conditions	Bacterial count	Increased sulfamase activity but no significant taxonomic change	(Mallett et al. 1985)
	Acesulfame K		ND	ND	Neither antibacterial effects nor promotion of bacterial growth were observed	(Rymon Lipinski 1985)
	Sucralose		Rats gavaged with 100, 300, 500, 1000 mg/kg bw/day slenda (containing 1.1% w/w sucralose + maltodextrin + glucose) for 12 weeks	ND	Number of total anaerobes, bifidobacteria, lactobacilli, *Bacteroides,* clostridia and total aerobic bacteria decreased in feces. No effect on enterobacteria.	(Abou-Donia et al. 2008)
	Saccharin		Mice treated with commercial formulation or pure saccharin in drinking water	16S rRNA gene sequencing + shotgun metagenomic sequencing	Fecal microbiota of saccharin-consuming mice displayed considerable dysbiosis, among which increased in bacteria from the Bacteroides genus and Clostridiales order. Glycan degradation pathway is over represented and SCFA (propionate and acetate) are elevated in stool from saccharin-consuming mice.	(Suez et al. 2014)

Compound			Experimental protocol			
**Class**	**Name**	**Use**	**Exposure protocol**	**Parameters evaluated**	**Results**	**Reference**
Miscellaneous	Holoacetic acids	By-products of drinking water disinfection	Rats provided water ad libitum containing 1g/L BCA, DCA or DBA for up to one month	Bacterial count	Growth inhibition of caecal bacteria , especially enterococci	(George et al. 2000)
				Enzyme activity	BCA: caecal beta-galactosidase activity elevated, large intestinal beta-galactosidase and beta-glucosidase elevated, cecal azoreductase decreased DCA: cecal beta-galactosidase and beta-glucuronidase activity decreased. large intestinal beta-galactosidase and beta-glucosidase elevated, cecal azoreductase, nitroreductase and dechlorinase decreased. DBA: ceacal beta-galactosidase and beta-glucuronidase activity decreased. Large intestinal beta-glucosidase elevated, cecal azoreductase and dechlorinase decreased	
			Rat caecal homogenates incubate anaerobically with BCA	Enzyme activity	Increase in beta-glucosidase and dehydrochlorinase activities.	(Nelson et al. 2001)
	Nonylphenol	Plasticizer	*L. acidophilus* and *B. bifidum*	Bacterial growth	Dose dependent growth inhibition of *L. acidophilus* and *B. bifidum*	(Hsu et al. 2009)
	TCDD	Dioxin	Mice	Bacterial count	No change in bacterial count	(Ishikawa 2009)
	Particulate matter	Arise from vehicle exhaust and industrial emissions	Mice gavaged with Ottawa urban PM10 for 35 days	Bacterial composition	Significant changes in amounts of Bacteroides, Firmicutes and Verrucomicrobia bacteria in feces.	(Kish et al. 2013)
				Cecal SCFA	Decrease in butyric acid and valeric acid in cecum.	
	Sudan dyes	Colorants in food products	11 prevalent human intestinal bacterial strains	Bacterial growth	Cell growth of 2, 3, 5, 5, and 1 strains inhibited by Sudan I, II, III, IV and Para Red resp.	(Pan et al. 2012)
	PCBs	Were widely used as dielectric and coolant fluids	Mice treated by oral gavage with a mixture of PCB153, PCB138, and PCB180 (150 μmol/kg) for 2 days	16S rRNA	Decreased abundance of 1,133 taxa, primarily Proteobacteria.	(Choi et al. 2013)
	2,3,7,8-tetrachlorodi benzofuran (TCDF)	Persistent organic pollutant	WT and AhR-/- mice fed a diet containing 24 mg/kg for 5 days	16S rRNA gene sequencing + metabolomics	TCDF-treated mouse cecal contents enriched with *Butyrivibrio sp*. but depleted in *Oscillobacter sp*. Bile acid metabolism, hepatic lipogenesis, glucogenesis and glycogenolysis altered in an AhR-dependant manner.	(Zhang et al. 2015)

References:

Abou-Donia MB, El-Masry EM, Abdel-Rahman AA, McLendon RE, Schiffman SS. 2008. Splenda alters gut microflora and increases intestinal p-glycoprotein and cytochrome p-450 in male rats. J. Toxicol. Environ. Health Part A 71:1415–1429; doi:10.1080/15287390802328630.

Bakke J, Struble C, Gustafsson JA, Gustafsson B. 1985. Catabolism of premercapturic acid pathway metabolites of naphthalene to naphthols and methylthio-containing metabolites in rats. Proc Natl Acad Sci USA 82: 668–671.

Bakke JE, Gustafsson JA, Gustafsson BE. 1980. Metabolism of propachlor by the germfree rat. Science 210: 433–435.

Ball LM, Rafter JJ, Gustafsson JA, Gustafsson BE, Kohan MJ, Lewtas J. 1991. Formation of mutagenic urinary metabolites from 1-nitropyrene in germ-free and conventional rats: role of the gut flora. Carcinogenesis 12: 1–5.

Ball LM, Renwick AG, Williams RT. 1977. The fate of [14C]saccharin in man, rat and rabbit and of 2-sulphamoyl[14C]benzoic acid in the rat. Xenobiotica 7: 189–203.

Bickel MH, Burkard B, Meier-Strasser E, van den Broek-Boot M. 1974. Entero-bacterial formation of cyclohexylamine in rats ingesting cyclamate. Xenobiotica 4: 425–439.

Brandt I, Klasson-Wehler E, Rafter J, Bergman A. 1982. Metabolism of 2,4",5-trichlorobiphenyl: tissue concentrations of methylsulphonyl-2,4,"5-trichlorobiphenyl in germfree and conventional mice. Toxicol Lett 12: 273–280.

Breton J, Massart S, Vandamme P, De Brandt E, Pot B, Foligné B. 2013. Ecotoxicology inside the gut: impact of heavy metals on the mouse microbiome. BMC Pharmacol Toxicol 14:62; doi:10.1186/2050-6511-14-62.

Bu N, Wang HY, Hao WH, Liu X, Xu S, Wu B, et al. 2011. Generation of thioarsenicals is dependent on the enterohepatic circulation in rats. Metallomics 3:1064–1073; doi:10.1039/c1mt00036e.

Cerniglia CE, Howard PC, Fu PP, Franklin W. 1984. Metabolism of nitropolycyclic aromatic hydrocarbons by human intestinal microflora. Biochem. Biophys. Res. Commun. 123: 262–270.

Choi JJ, Eum SY, Rampersaud E, Daunert S, Abreu MT, Toborek M. 2013. Exercise Attenuates PCB-Induced Changes in the Mouse Gut Microbiome. Environ Health Perspect; doi:10.1289/ehp.1306534.

Doolittle DJ, Sherrill JM, Butterworth BE. 1983. Influence of intestinal bacteria, sex of the animal, and position of the nitro group on the hepatic genotoxicity of nitrotoluene isomers in vivo. Cancer Res. 43: 2836–2842.

Drasar BS, Renwick AG, Williams RT. 1972. The role of the gut flora in the metabolism of cyclamate. Biochem. J. 129: 881–890.

El-Bayoumy K, Sharma C, Louis YM, Reddy B, Hecht SS. 1983. The role of intestinal microflora in the metabolic reduction of 1-nitropyrene to 1-aminopyrene in conventional and germfree rats and in humans. Cancer Lett. 19: 311–316.

Fazeli M, Hassanzadeh P, Alaei S. 2011. Cadmium chloride exhibits a profound toxic effect on bacterial microflora of the mice gastrointestinal tract. Hum Exp Toxicol 30:152–159; doi:10.1177/0960327110369821.

Fu PP, Cerniglia CE, Richardson KE, Heflich RH. 1988. Nitroreduction of 6-nitrobenzo[a]pyrene: a potential activation pathway in humans. Mutat. Res. 209: 123–129.

George SE, Nelson GM, Swank AE, Brooks LR, Bailey K, George M, et al. 2000. The disinfection by-products dichloro-, dibromo-, and bromochloroacetic acid impact intestinal microflora and metabolism in Fischer 344 rats upon exposure in drinking water. Toxicol Sci 56: 282–289.

Guest D, Schnell SR, Rickert DE, Dent JG. 1982. Metabolism of 2,4-dinitrotoluene by intestinal microorganisms from rat, mouse, and man. Toxicol Appl Pharmacol 64: 160–168.

Gustafsson JA, Rafter JJ, Bakke JE, Gustafsson BE. 1981. The effect of intestinal microflora on the enterohepatic circulation of mercapturic acid pathway metabolites. Nutr Cancer 2:224–231; doi:10.1080/01635588109513687.

Howard PC, Beland FA, Cerniglia CE. 1983. Reduction of the carcinogen 1-nitropyrene to 1-aminopyrene by rat intestinal bacteria. Carcinogenesis 4: 985–990.

Hsu WT, Lin TH, Chang EE, Hung CH, Huang AL, Wu TC, et al. 2009. The effect of nonylphenol on the growth of Lactobacillus acidophilus and Bifidobacterium bifidum. J Microbiol Immunol Infect 42: 451–456.

Ishikawa S. 2009. Children's immunology, what can we learn from animal studies (3): Impaired mucosal immunity in the gut by 2,3,7,8-tetraclorodibenzo-p-dioxin (TCDD): a possible role for allergic sensitization. J Toxicol Sci 34 Suppl 2: SP349–61.

Joly C, Gay-Quéheillard J, Léké A, Chardon K, Delanaud S, Bach V, et al. 2013. Impact of chronic exposure to low doses of chlorpyrifos on the intestinal microbiota in the Simulator of the Human Intestinal Microbial Ecosystem (SHIME) and in the rat. Environ Sci Pollut Res Int 20:2726–2734; doi:10.1007/s11356-012-1283-4.

Kataoka K, Kinouchi T, Akimoto S, Ohnishi Y. 1995. Bioactivation of cysteine conjugates of 1-nitropyrene oxides by cysteine conjugate beta-lyase purified from Peptostreptococcus magnus. Appl Environ Microbiol 61: 3781–3787.

Khanal T, Kim HG, Choi JH, Do MT, Kong MJ, Kang MJ, et al. 2012. Biotransformation of geniposide by human intestinal microflora on cytotoxicity against HepG2 cells. Toxicol Lett 209:246–254; doi:10.1016/j.toxlet.2011.12.017.

King LC, Kohan MJ, George SE, Lewtas J, Claxton LD. 1990. Metabolism of 1-nitropyrene by human, rat, and mouse intestinal flora: mutagenicity of isolated metabolites by direct analysis of HPLC fractions with a microsuspension reverse mutation assay. J Toxicol Environ Health 31:179–192; doi:10.1080/15287399009531447.

Kinouchi T, Kataoka K, Miyanishi K, Akimoto S, Ohnishi Y. 1992. Role of intestinal microflora in metabolism of glutathione conjugates of 1-nitropyrene 4,5-oxide and 1-nitropyrene 9,10-oxide. Tohoku J. Exp. Med. 168: 119–122.

Kinouchi T, Morotomi M, Mutai M, Fifer EK, Beland FA, Ohnishi Y. 1986. Metabolism of 1-nitropyrene in germ-free and conventional rats. Jpn. J. Cancer Res. 77: 356–369.

Kish L, Hotte N, Kaplan GG, Vincent R, Tso R, Gänzle M, et al. 2013. Environmental particulate matter induces murine intestinal inflammatory responses and alters the gut microbiome. PLoS ONE 8:e62220; doi:10.1371/journal.pone.0062220.

Krüger M, Shehata AA, Schrödl W, Rodloff A. 2013. Glyphosate suppresses the antagonistic effect of Enterococcus spp. on Clostridium botulinum. Anaerobe 20:74–78; doi:10.1016/j.anaerobe.2013.01.005.

Levin AA, Dent JG. 1982. Comparison of the metabolism of nitrobenzene by hepatic microsomes and cecal microflora from Fischer-344 rats in vitro and the relative importance of each in vivo. Drug Metab. Dispos. 10: 450–454.

Lu K, Abo RP, Schlieper KA, Graffam ME, Levine S, Wishnok JS, et al. 2014. Arsenic exposure perturbs the gut microbiome and its metabolic profile in mice: an integrated metagenomics and metabolomics analysis. Environ Health Perspect 122:284–291; doi:10.1289/ehp.1307429.

Macholz R, Kujawa M, Schulze J, Lewerenz HJ, Schnaak W. 1985. The metabolism of some xenobiotics in germ-free and conventional rats. Arch. Toxicol. Suppl. 8: 373–376.

Mallett AK, Rowland IR, Bearne CA, Purchase R, Gangolli SD. 1985. Metabolic adaptation of rat faecal microflora to cyclamate in vitro. Food Chem Toxicol 23: 1029–1034.

Manning BW, Campbell WL, Franklin W, Delclos KB, Cerniglia CE. 1988. Metabolism of 6-nitrochrysene by intestinal microflora. Appl Environ Microbiol 54: 197–203.

Manning BW, Cerniglia CE, Federle TW. 1986. Biotransformation of 1-nitropyrene to 1-aminopyrene and N-formyl-1-aminopyrene by the human intestinal microbiota. J Toxicol Environ Health 18:339–346; doi:10.1080/15287398609530875.

Mendel JL, Walton MS. 1966. Conversion of p,p“ -DDT to p,p” -DDD by intestinal flora of the rat. Science 151: 1527–1528.

Michalke K, Schmidt A, Huber B, Meyer J, Sulkowski M, Hirner AV, et al. 2008. Role of intestinal microbiota in transformation of bismuth and other metals and metalloids into volatile methyl and hydride derivatives in humans and mice. Appl Environ Microbiol 74:3069–3075; doi:10.1128/AEM.02933-07.

Mirsalis JC, Hamm TE, Sherrill JM, Butterworth BE. 1982. Role of gut flora in the genotoxicity of dinitrotoluene. Nature 295: 322–323.

Möller L, Corrie M, Midtvedt T, Rafter J, Gustafsson JA. 1988. The role of the intestinal microflora in the formation of mutagenic metabolites from the carcinogenic air pollutant 2-nitrofluorene. Carcinogenesis 9: 823–830.

Möller L, Zeisig M, Midtvedt T, Gustafsson JA. 1994. Intestinal microflora enhances formation of DNA adducts following administration of 2-NF and 2-AAF. Carcinogenesis 15: 857–861.

Nakamura I, Hosokawa K, Tamura H, Miura T. 1977. Reduced mercury excretion with feces in germfree mice after oral administration of methyl mercury chloride. Bull Environ Contam Toxicol 17: 528–533.

Nelson GM, Swank AE, Brooks LR, Bailey KC, George SE. 2001. Metabolism, microflora effects, and genotoxicity in haloacetic acid-treated cultures of rat cecal microbiota. Toxicol Sci 60: 232–241.

Palmnäs MSA, Cowan TE, Bomhof MR, Su J, Reimer RA, Vogel HJ, et al. 2014. Low-dose aspartame consumption differentially affects gut microbiota-host metabolic interactions in the diet-induced obese rat. PLoS ONE 9:e109841; doi:10.1371/journal.pone.0109841.

Pan H, Feng J, He G-X, Cerniglia CE, Chen H. 2012. Evaluation of impact of exposure of Sudan azo dyes and their metabolites on human intestinal bacteria. Anaerobe 18:445–453; doi:10.1016/j.anaerobe.2012.05.002.

Philbert MA, Gray AJ, Connors TA. 1987. Preliminary investigations into the involvement of the intestinal microflora in CNS toxicity induced by 1,3-dinitrobenzene in male F-344 rats. Toxicol Lett 38: 307–314.

Rafil F, Franklin W, Heflich RH, Cerniglia CE. 1991. Reduction of nitroaromatic compounds by anaerobic bacteria isolated from the human gastrointestinal tract. Appl Environ Microbiol 57: 962–968.

Reddy BG, Pohl LR, Krishna G. 1976. The requirement of the gut flora in nitrobenzene-induced methemoglobinemia in rats. Biochem Pharmacol 25: 1119–1122.

Renwick AG, Williams RT. 1969. Gut bacteria and the metabolism of cyclamate in the rat. Biochem. J. 114: 78P.

Renwick AG, Williams RT. 1972. The fate of cyclamate in man and other species. Biochem. J. 129: 869–879.

Richardson KE, Fu PP, Cerniglia CE. 1988. Metabolism of 1-, 3-, and 6-nitrobenzo[a]pyrene by intestinal microflora. J Toxicol Environ Health 23:527–537; doi:10.1080/15287398809531134.

Rickert DE, Long RM, Krakowka S, Dent JG. 1981. Metabolism and excretion of 2,4-[14C]Dinitrotoluene in conventional and axenic Fischer-344 rats. Toxicol Appl Pharmacol 59: 574–579.

Rowland IR, Davies MJ, Evans JG. 1980. Tissue content of mercury in rats given methylmercuric chloride orally: influence of intestinal flora. Arch. Environ. Health 35: 155–160.

Rowland IR, Davies MJ, Grasso P. 1978. Metabolism of methylmercuric chloride by the gastro-intestinal flora of the rat. Xenobiotica 8: 37–43.

Rymon Lipinski von G-W. 1985. The New Intense Sweetener Acesulfame K. Food Chemistry 16: 259–269.

Seko Y, Miura T, Takahashi M, Koyama T. 1981. Methyl mercury decomposition in mice treated with antibiotics. Acta Pharmacol Toxicol (Copenh) 49: 259–265.

Shehata AA, Schrödl W, Aldin AA, Hafez HM, Krüger M. 2013. The effect of glyphosate on potential pathogens and beneficial members of poultry microbiota in vitro. Curr. Microbiol. 66:350–358; doi:10.1007/s00284-012-0277-2.

Stein K, Portig J, Fuhrmann H, Koransky W, Noack G. 1980. Steric factors in the pharmacokinetics of lindane and alpha-hexachlorocyclohexane in rats. Xenobiotica 10: 65–77.

Suez J, Korem T, Zeevi D, Zilberman-Schapira G, Thaiss CA, Maza O, et al. 2014. Artificial sweeteners induce glucose intolerance by altering the gut microbiota. Nature 514:181–186; doi:10.1038/nature13793.

Van de Wiele T, Gallawa CM, Kubachka KM, Creed JT, Basta N, Dayton EA, et al. 2010. Arsenic metabolism by human gut microbiota upon in vitro digestion of contaminated soils. Environ Health Perspect 118:1004–1009; doi:10.1289/ehp.0901794.

Van de Wiele T, Vanhaecke L, Boeckaert C, Peru K, Headley J, Verstraete W, et al. 2005. Human colon microbiota transform polycyclic aromatic hydrocarbons to estrogenic metabolites. Environ Health Perspect 113: 6–10.

Xu H, Heinze TM, Chen S, Cerniglia CE, Chen H. 2007. Anaerobic metabolism of 1-amino-2-naphthol-based azo dyes (Sudan dyes) by human intestinal microflora. Appl Environ Microbiol 73:7759–7762; doi:10.1128/AEM.01410-07.

Xu H, Heinze TM, Paine DD, Cerniglia CE, Chen H. 2010. Sudan azo dyes and Para Red degradation by prevalent bacteria of the human gastrointestinal tract. Anaerobe 16:114–119; doi:10.1016/j.anaerobe.2009.06.007.

Yim Y-J, Seo J, Kang S-I, Ahn J-H, Hur H-G. 2008. Reductive dechlorination of methoxychlor and DDT by human intestinal bacterium Eubacterium limosum under anaerobic conditions. Arch. Environ. Contam. Toxicol. 54:406–411; doi:10.1007/s00244-007-9044-y.

Zhang L, Nichols RG, Correll J, Murray IA, Tanaka N, Smith PB, et al. 2015. Persistent Organic Pollutants Modify Gut Microbiota-Host Metabolic Homeostasis in Mice Through Aryl Hydrocarbon Receptor Activation. Environ Health Perspect 123:679–688; doi:10.1289/ehp.1409055.

Zheng XX, Zhao AA, Xie GG, Chi YY, Zhao LL, Li HH, et al. 2013. Melamine-induced renal toxicity is mediated by the gut microbiota. Sci Transl Med 5:172ra22–172ra22; doi:10.1126/scitranslmed.3005114.
